# A Temperature-Precipitation Based Leafing Model and Its Application in Northeast China

**DOI:** 10.1371/journal.pone.0033192

**Published:** 2012-04-11

**Authors:** Rong-Ping Li, Guang-Sheng Zhou

**Affiliations:** 1 State Key Laboratory of Vegetation and Environmental Change, Institute of Botany, Chinese Academy of Sciences, Beijing, China; 2 Graduate School of Chinese Academy of Sciences, Beijing, China; 3 Institute of Atmospheric Environment, China Meteorological Administration, Shenyang, China; 4 Chinese Academy of Meteorological Sciences, Beijing, China; University of Washington, United States of America

## Abstract

Plant phenology models, especially leafing models, play critical roles in evaluating the impact of climate change on the primary production of temperate plants. Existing models based on temperature alone could not accurately simulate plant leafing in arid and semi-arid regions. The objective of the present study was to test the suitability of the existing temperature-based leafing models in arid and semi-arid regions, and to develop a temperature-precipitation based leafing model (TP), based on the long-term (i.e., 12–27 years) ground leafing observation data and meteorological data in Northeast China. The better simulation of leafing for all the plant species in Northeast China was given by TP with the fixed starting date (TPn) than with the parameterized starting date (TPm), which gave the smallest average root mean square error (*RMSE*) of 4.21 days. Tree leafing models were validated with independent data, and the coefficient of determination (*R* ^2^) was greater than 0.60 in 75% of the estimates by TP and the spring warming model (SW) with the fixed starting date. The average *RMSE* of herb leafing simulated by TPn was 5.03 days, much lower than other models (>9.51 days), while the average *R* ^2^ of TPn and TPm were 0.68 and 0.57, respectively, much higher than the other models (<0.22). It indicates that TPn is a universal model and more suitable for simulating leafing of trees and herbs than the prior models. Furthermore, water is an important factor determining herb leafing in arid and semi-arid temperate regions.

## Introduction

Plant leafing and yellowing stages both play critical roles in accurately estimating carbon and water flux exchanges between the land and atmosphere [1], [2] and the changes in land surface characteristics [3], [4]. Moreover, plant leafing is more sensitive to climate change than plant yellowing [5], [6]. The ability to precisely predict plant leafing is crucial to modeling the impacts of climate change on plant primary productivity.

Currently, there are many phenological models to predict the changes in plant spring phenology, including bud, leafing, and flowering stages [7]–[12]. The simplest spring phenological models consider only temperature, as exemplified by the cumulated temperature model [13], [14]. More complex models based on the intensive study of plant physiology incorporate the dual roles of temperature (i.e., chilling and forcing); such models include the sequential model [9], [12], [15], the parallel model [9], [10], [16], and the alternating model [7], [17]. The most complex phenology models consider the impact of day length as well as temperature, for example, the light and temperature phenology model [10]. Overall, the current temperature-based spring phenology models are mainly used for tree species in temperate humid and semi-humid areas; the efficacy of these models for simulating spring phenology for trees and herbs in temperate arid and semi-arid regions is untested.

Precipitation is also a key determinant of plant leafing, especially in semi-arid and arid area, however, the impact of precipitation on spring phenology has seldom been considered in plant leafing models. Yuan et al. [18] initially explored the leafing responses of dominant herbs (*Leymus chinensis* and *Stipa grandis*) to soil moisture in Inner Mongolia and developed a leafing model for *L. chinensis* and *S. grandis* based on the effects of temperature and soil moisture. However, the model was established only for two herbs and soil moisture is rarely measured. This model, moreover, was not validated by other external data [18].

Northeast China, located at high latitude in the northern hemisphere, is highly sensitive to climate change and has experienced the increase in air temperature twice that of the global average [19]. Furthermore, precipitation varies significantly in Northeast China. There exists a latitude-based thermal gradient, including warm (south), moderate, and cold (north) temperate regions. From east to west, there are various precipitation zones, including humid, semi-humid and semi-arid regions. Thus, precise modeling of tree and herb leafing in Northeast China is rather complicated, yet it is crucial to simulate the environmental consequences of climate change in this region.

The present study is based on long-term (i.e., 12–27 years) ground observations of leafing and simple meteorological data (i.e., daily mean temperature and precipitation). The leafing response of 13 plants dominant in Northeast China (including eight trees and five herbs) to hydrothermal factors would be evaluated and simulated. Our main objectives were to determine: (1) whether the existing temperature-based models accurately simulate tree and herb leafing in arid and semi-arid regions; and (2) whether a temperature-precipitation based leafing model can more precisely simulate plant leafing under different water and heat conditions in arid and semi-arid regions.

## Methods

### Study site and plant species

**Figure 1:**
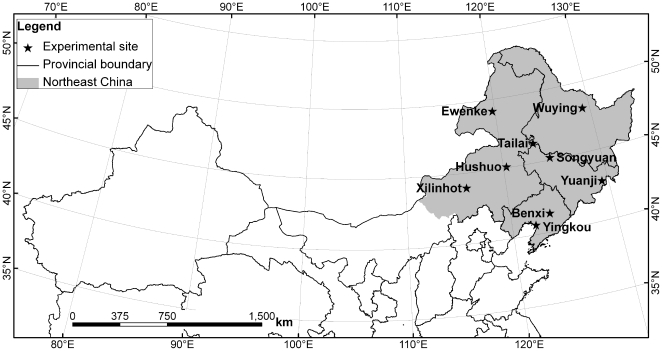
Locations of the study area and nine Agricultural Meteorological Experiment Stations in Northeast China.

Northeast China consists of Heilongjiang, Jilin, Liaoning provinces and four leagues of Inner Mongolia (Fig. 1). The region has a continental monsoon climate. Mean annual air temperature is 4.5°C, with an average temperature of −18.2°C in the coldest month (January), and an average of 22.4°C in the warmest month (July). Annual average precipitation is 514 mm, 77% of which falls from May to August. The region has a minimum annual precipitation of 245 mm in the west, a typical semi-arid area, and a maximum annual precipitation of 1079 mm in the east.

In the east of Northeast China, Great Xing'an Mountains is the cold temperate coniferous forest zone, dominated by typical tree species *Larix dahurica*. Changbai mountains, typical temperate coniferous and broadleaved mixed forest zone, dominated by *Pinus koraiensis*. The west of Northeast China is the typical zone of temperate steppe with herbs such as *Leymus chinensis*, *Stipa krylovii*, *Agropyron cristatum* and *S. baicalensis* [20]. In Northeast China, *Populus simonii* is the main afforestation tree species, and *Salix matsudana*, *Armeniaca vulgaris*, and *Ulmus pumila* are the common garden species. These plant species are strictly controlled by hydrothermal conditions. Thus, thirteen dominant plant species are selected in the present study, including eight trees (*Salix matsudana*, *Armeniaca vulgaris*, *Ulmus pumila*, *Populus simonii*, *Syringa oblate*, *Pinus koraiensis*, *Larix dahurica*, and *Picea koraiensis*) and five herbs (*Leymus chinensis*, *Stipa krylovii*, *S. baicalensis*, *Elymus dahuricus*, and *Agropyron cristatum*) (Table 1).

**Table 1:** Observation sites in Northeast China and the plant species studied for leafing at those sites.

Location	Province	Longitude (°E)	Latitude (°N)	Elevation (m)	Species	Observation period (year)
Benxi	Liaoning	123.78	41.32	182.5	*Salix matsudana*	1981–2005
Yingkou	Liaoning	122.27	40.67	4.3	*Syringa oblate*	1981–2005
Songyuan	Jilin	124.83	45.18	139.7	*Populus simonii*	1981–2005
Yuanji	Jilin	129.40	42.77	240.6	*Armeniaca vulgaris*	1984–2005
Tailai	Heilongjiang	123.42	46.40	149.5	*Ulmus pumila*	1982–2004
Wuying	Heilongjiang	129.25	48.12	299.1	*Pinus koraiensis Larix dahurica Picea koraiensis*	1991–2002 1991–2002 1991–2002
Xilinhot	Inner Mongolia	116.07	43.95	991	*Leymus chinensis Stipa krylovii*	1985–2004 1985–2004
Ewenke	Inner Mongolia	119.75	49.15	621	*Leymus chinensis Stipa baicalensis*	1986–2006 1986–2006
Hushuo	Inner Mongolia	120.33	45.07	629	*Elymus dahuricus Agropyron cristatum*	1980–2006 1988–2006

### Phenological data collection

The leafing data of dominant plants were collected from nine Agricultural Meteorological Experiment Stations, China Meteorological Administration, located in Northeast China. Plant leafing status was observed daily; plants were considered to have leafed if: (1) the first flat leaf had appeared from the buds of trees with simple leaves; (2) young leaves had emerged from the leaf sheaths of conifers; (3) one or two leaflets of compound leaves had unfolded; or (4) old exposed leaves of over-wintering herbs had turned from yellow to green, and the first leaf of herbs had emerged above the ground [21].

### Meteorological data collection

Meteorological data, including daily mean temperature, daily precipitation, daily minimum temperature and relative humidity, were collected from nine Agriculture Meteorological Stations where plant leafing was observed.

### Phenology model

Generally, temperature is considered to be the main driving factor of plant leafing. Representative temperature-based phenology models include spring warming model (SW) [14], sequential model (SM) [9], [10], [22], parallel model (PM) [9], [10], [23], and alternating model (AM) [8], [10], [23]. These four models were used to simulate leafing in the present study. Their equations are summarized in Table 2 [23].

**Table 2:** Equations of the phenology models compared in the present study.

Model type	Equation of models
Spring warming model (SW)	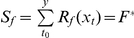
	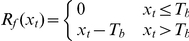
Sequential model (SM)	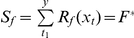
	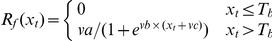
	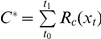
	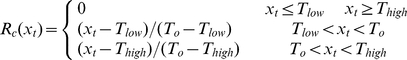
Parallel model (PM)	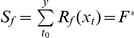
	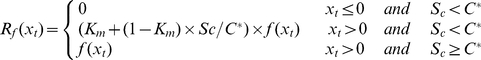
	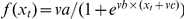
	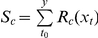
	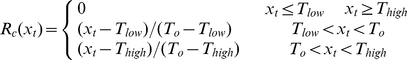
Alternating model (AM)	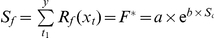
	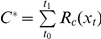
	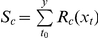
	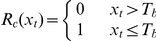
	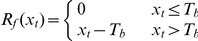
Growth season index (GSI)	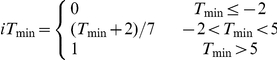
	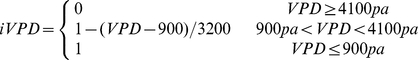
	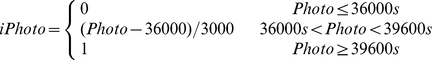
	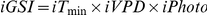

*y*: date of leafing; *x_t_*: daily mean air temperature in degrees Celsius; *R_f_(x_t_)*: forcing rate function; *R_c_(x_t_)*: chilling rate function; *S_f_*: state of forcing; *S_c_*: state of chilling; *K_m_*: minimum potential of unchilled buds to respond to forcing temperature; C*: critical value of state of chilling for the transition from rest to quiescence; F*: temperature sum critical threshold; *t_0_*: starting day of the heat sum calculation or date of onset of rest; *t_1_*: date of onset of quiescence; *T_b_*: base temperature; *T_o_*: optimal temperature of the rate of chilling; *T_low_*: the lowest temperature of the rate of chilling; *T_high_*: the highest temperature of the rate of chilling; *a*, *b*, *va*, *vb* and *vc*: constants; *iT_min_*: daily indicator for minimum temperature; *T_min_*: observed daily minimum temperature in degrees Celsius; *iVPD*: daily indicator for vapor pressure deficit; *VPD*: observed daily vapor pressure deficit in Pascals; *iPhoto*: daily photoperiod indicator; *Photo*: daily photoperiod in seconds; *iGSI*: daily growing season index.

Water plays a critical role in regulating plant phenology in arid and semi-arid areas [18], [24], [25], and has been included in some phenology models. Examples include cumulative precipitation in the current year [26], vapor pressure deficit in the growth season index (GSI) [25], and soil moisture [18]. However, the combination of hydrological and thermal factors has not been considered. Usually, when both hydrological and thermal conditions reach certain thresholds, plants begin leafing. Previous studies of plant leafing phenology [18], [24] have identified the accumulated precipitation in the previous year and current year as an important hydrological factor and the accumulated temperature in the current year as an important thermal factor affecting plant leafing. Thus, a new plant leafing model (so-called TP) based on the effects of both temperature and precipitation could be expressed as:
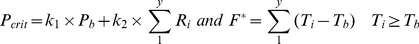
(1)where *k_1_*, *k_2_*, *P_crit_*, *T_b_*, and F^*^ are parameters obtained through optimization. *k_1_*, *k_2_* are the efficiency of precipitation in the previous and current (prior to leafing) year in affecting leafing in the current year. *P_b_* is the annual precipitation in the previous year. *y* is the day of plant leafing in the current year. *R_i_* is the daily precipitation in the current year (mm). *P_crit_* is the water threshold (mm). *T_i_* is the average daily temperature (°C) in the current year. *T_b_* is the base temperature (°C). F^*^ is the temperature sum critical threshold (°C). Models should always be validated with independent data not used to construct the model itself [27]. In this study, the phenology model parameters were estimated using leafing data from odd-numbered years (12 years), and the simulation accuracy was tested with the independent even-year data (12 years).

### Parameter estimation

Phenological data were converted to Julian day (DOY). Model parameters were estimated using the least root mean square error (*RMSE*) method:
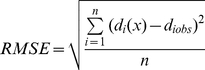
(2)where *d_i_*(*x*) is the predicted date of plant leafing in the *i*th year and *d_iobs_* is the observed value of plant leafing in the *i*th year. The model was evaluated by *F*-tests. The optimized parameters of the model were determined using the simulated annealing method [8]. The average *RMSE*, coefficient of determination (*R* ^2^) were used to validate the model in the present study.

Using odd-year data, the parameter sets and three statistic variables (*RMSE*, *R^2^* and *F*) were given in Tables 3, 4, 5 for five models (i.e., SW, SM, PM, AM, and TP) (Table 2). Both fixed and parameterized starting date were considered for SW, TP and AM, i.e., the fixed starting date was set on 1 January for SW and TP and 1 September for AM. The starting date (*t*_0_), the minimum (*T*_low_) and maximum (*T*_high_) values of chill temperature, and the parameters values (*va*, *vb*, *vc*) of response curves for forcing temperature were fixed (e.g., *t*_0_, *T*_low_, *T*_high_, *va*, *vb* and *vc* were set to September 1, −3.4, 10.4, 28.4, −0.185 and −18.4, respectively) and parameterized for SM and PM, based on the reference and parameterization in the present study.

**Table 3:** Parameter values of spring warming model (SW), temperature-precipitation based leafing model (TP), and alternating model (AM) for plant leafing in Northeast China.

Species		SW			TP						AM				
		*t*_0_	*T*_b_ (°C)	*F* ^*^(°C day)	*t*_0_	*T*_b_ (°C)	*F* ^*^(°C day)	*k*_1_	*k*_2_	*P_crit_* (mm)	*t*_0_	*T*_b_ (°C)	*C* ^*^(CU)	*a*	*b*
PSM	m	Apr.12	0.6	36.8	Apr. 12	7.4	6.2	0.044	0.669	28.49	Nov. 8	0.1	2.13	215.2	−0.0431
	n	**Jan. 1**	0.2	209.0	**Jan. 1**	6.2	53.3	0.013	0.040	20.34	**Sep. 1**	0.0	28.47	835.2	0.05
PUP	m	Apr.10	1.3	199.2	Apr 11	2.8	150.6	0.018	0.216	9.63	Oct. 7	8.8	1.32	52.03	−0.0385
	n	**Jan. 1**	0.9	271.6	**Jan. 1**	0.2	284.1	0.044	0.650	9.12	**Sep. 1**	7.2	2.13	85.37	−0.02
PAV	m	Mar.30	7.0	69.4	Mar. 20	7.0	68.7	0.099	0.579	21.48	Sep. 14	7.6	3.39	53.06	−0.0013
	n	**Jan. 1**	7.0	68.6	**Jan. 1**	3.0	176.5	0.054	0.088	31.64	**Sep. 1**	7.3	3.30	55.90	−0.03
PPS	m	Mar.22	0.0	280.6	Mar. 30	0.1	247.9	0.088	0.450	26.29	Oct. 4	0.5	11.52	353.5	−0.0269
	n	**Jan. 1**	3.3	178.3	**Jan. 1**	5.7	110.7	0.095	0.800	16.75	**Sep. 1**	0.0	5.23	299.0	−0.01
PSO	m	Apr.1	9.1	25.7	Apr. 11	9.0	26.1	0.015	0.849	4.92	Feb. 24	7.5	9.74	62.2	−0.0241
	n	**Jan. 1**	0.0	288.6	**Jan. 1**	2.1	210.2	0.096	0.993	21.38	**Sep. 1**	9.0	9.16	46.5	−0.01
PPNK	m	May29	0.0	554.2	Jun. 8	2.4	325.4	0.012	0.120	23.18	Sep. 3	14.6	0.21	107.8	−0.0323
	n	**Jan. 1**	1.2	903.3	**Jan. 1**	9.3	293.6	0.010	0.179	34.04	**Sep. 1**	14.6	0.93	115.1	0.08
PLD	m	May1	4.7	123.1	May 1	5.4	107.0	0.034	0.403	16.58	Sep. 8	4.3	0.52	200.7	−0.0812
	n	**Jan. 1**	3.1	217.3	**Jan. 1**	9.8	38.69	0.023	0.447	13.33	**Sep. 1**	5.7	1.77	151.2	−0.04
PPCK	m	May 11	9.2	67.7	Apr. 28	1.0	325.3	0.023	0.295	13.67	Sep. 25	7.4	0.28	135.6	−0.0462
	n	**Jan. 1**	2.1	352.1	**Jan. 1**	0.5	468.0	0.049	0.073	22.66	**Sep. 1**	7.4	4.65	161.6	−0.04
XLC	m	Apr.16	2.3	16.1	Mar. 19	2.2	45.4	0.076	0.158	15.48	Mar. 26	2.4	4.76	52.17	−0.0683
	n	**Jan. 1**	1.1	83.9	**Jan. 1**	2.3	44.3	0.073	0.168	15.18	**Sep. 1**	1.6	2.45	99.28	−0.09
XSK	m	Apr.11	3.3	6.0	Mar. 20	0.5	52.6	0.079	0.094	14.84	Oct. 4	0.0	0.64	109.3	−0.0818
	n	**Jan. 1**	4.1	19.7	**Jan. 1**	0.1	58.4	0.097	0.104	18.09	**Sep. 1**	1.6	1.85	53.25	−0.01
ELC	m	Apr.10	0.2	82.9	Jan. 17	0.0	108.1	0.052	0.166	12.61	Oct. 17	0.3	5.06	146.9	−0.0951
	n	**Jan. 1**	0.5	98.5	**Jan. 1**	0.1	101.3	0.040	0.699	15.76	**Sep. 1**	4.6	21.33	58.0	−0.01
ESB	m	Apr.11	0.4	84.0	Mar. 17	0.0	115.2	0.097	0.154	18.03	Oct. 5	0.8	0.42	102.4	−0.0831
	n	**Jan. 1**	0.0	109.4	**Jan. 1**	0.1	114.3	0.024	0.771	8.63	**Sep. 1**	0.1	30.6	168.0	−0.02
HAC	m	Apr.9	7.3	12.4	Mar. 25	1.5	67.5	0.035	0.702	14.89	Mar. 23	7.1	12.43	28.8	−0.071
	n	**Jan. 1**	1.5	92.3	**Jan. 1**	1.5	64.7	0.036	0.847	16.00	**Sep. 1**	5.6	14.13	40.6	−0.04
HED	m	Apr.12	3.0	45.2	Apr. 9	6.7	7.9	0.046	0.937	19.54	Mar.11	1.6	10.54	96.2	−0.0174
	n	**Jan. 1**	1.1	101.1	**Jan. 1**	0.3	89.7	0.042	0.446	15.86	**Sep.1**	6.2	8.64	46.3	−0.08

m: parameterized starting date (*t_0_*); n: fixed starting date (*t_0_*); *T_b_*: base temperature; F^*^: temperature sum critical threshold; *k_1_*: efficiency of precipitation in the previous year in affecting leafing in the current year; *k_2_*: efficiency of precipitation in the current year (prior to leafing) in affecting leafing; CU: chilling unit; *P_crit_*: water threshold; C*: critical value of state of chilling for the transition from rest to quiescence; *a* and *b*: constants. PSM=*Salix matsudana*; PUP=*Ulmus pumila*; PAV=*Armeniaca vulgaris*; PPS=*Populus simonii*; PSO=*Syringa oblate*; PPNK=*Pinus koraiensis*; PLD=*Larix dahurica*; PPCK=*Picea koraiensis*; XLC=*Leymus chinensis* in Xilinhot; XSK=*Stipa krylovii*; ELC=*Leymus chinensis* in Ewenki; ESB=*Stipa baicalensis*; HAC=*Agropyron cristatum*; HED=*Elymus dahuricus*. The fixed parameters from Chuine et al. (1998) are in bold.

**Table 4:** Parameter values of sequential model (SM) and parallel model (PM) for plant leafing in Northeast China.

Species		SM										PM									
		*t_0_*	*T_b_* (°C)	C* (CU)	F^*^ (FU)	*T_o_* (°C)	*T_low_* (°C)	*T_high_* (°C)	*va*	*vb*	*vc*	*t_0_*	C* (CU)	F^*^ (FU)	*K_m_*	*T_o_* (°C)	*T_low_*(°C)	*T_high_* (°C)	*va*	*vb*	*vc*
PSM	m	Sep.22	5.9	5.6	50.3	5.7	5.5	11.1	40.0	−0.172	−25.8	Sep.3	28.7	265.8	0.05	6.2	−4.3	9.2	29.4	−0.094	−20.2
	n	**Sep.1**	1.6	9.3	126.8	7.9	**−3.4**	**10.4**	**28.4**	**−0.185**	**−18.4**	**Sep.1**	16.4	402.5	0.52	10.4	**−3.4**	**10.4**	**28.4**	**−0.185**	**−18.4**
PUP	m	Oct.8	11.7	0.7	9.1	7.5	1.4	11.7	1.1	−0.803	−5.6	Sep.2	1.0	468.2	0.28	4.9	0.2	8.5	33.0	−0.205	−15.9
	n	**Sep.1**	12.8	12.8	143.5	10.2	**−3.4**	**10.4**	**28.4**	**−0.185**	**−18.4**	**Sep.1**	13.3	361.2	0.47	10.3	**−3.4**	**10.4**	**28.4**	**−0.185**	**−18.4**
PAV	m	Sep.28	7.5	9.6	24.6	6.2	5.3	12.9	6.4	−0.101	−24.9	Dec.1	20.6	100.5	0.60	−4.0	−4.2	1.2	15.5	−0.113	−18.6
	n	**Sep.1**	6.1	10.0	119.5	10.4	**−3.4**	**10.4**	**28.4**	**−0.185**	**−18.4**	**Sep.1**	15.5	376.0	0.54	9.8	**−3.4**	**10.4**	**28.4**	**−0.185**	**−18.4**
PPS	m	Sep.8	10.2	8.9	30.8	8.4	−1.5	14.1	17.0	−0.127	−28.6	Sep.14	37.0	376.9	0.01	10.8	−4.4	11.0	60.1	−0.206	−17.5
	n	**Sep.1**	7.1	8.3	140.1	7.3	**−3.4**	**10.4**	**28.4**	**−0.185**	**−18.4**	**Sep.1**	8.7	329.0	0.31	9.8	**−3.4**	**10.4**	**28.4**	**−0.185**	**−18.4**
PSO	m	Feb.25	3.0	18.5	396.3	1.4	−4.6	13.8	48.8	−0.051	−20.5	Feb.4	15.6	484.2	0.13	5.5	−1.7	5.7	54.5	−0.366	−10.4
	n	**Sep.1**	10.0	8.4	88.3	9.3	**−3.4**	**10.4**	**28.4**	**−0.185**	**−18.4**	**Sep.1**	19.0	388.6	0.34	10.4	**−3.4**	**10.4**	**28.4**	**−0.185**	**−18.4**
PPNK	m	Nov.	6.8	9.3	23.3	4.6	4.3	11.0	31.9	−0.738	−25.2	Feb.19	11.2	570.9	0.01	12.5	4.2	13.0	21.1	−0.024	−15.7
	n	**Sep.1**	21.0	13.4	120.3	−2.6	**−3.4**	**10.4**	**28.4**	**−0.185**	**−18.4**	**Sep.1**	83.7	405.0	0.19	5.3	**−3.4**	**10.4**	**28.4**	**−0.185**	**−18.4**
PLD	m	Nov.13	8.7	13.8	26.0	6.6	−0.4	10.9	6.2	−0.159	−16.2	Apr.1	34.1	222.8	0.06	2.1	−4.8	15.0	23.3	−0.264	−10.2
	n	**Sep.1**	0.5	12.2	203.8	8.4	**−3.4**	**10.4**	**28.4**	**−0.185**	**−18.4**	**Sep.1**	0.9	381.4	0.58	10.1	**−3.4**	**10.4**	**28.4**	**−0.185**	**−18.4**
PPCK	m	Sep.22	8.1	2.1	44.2	4.2	−2.0	12.1	15.0	−0.178	−25.8	Sep.29	7.4	423.3	0.66	7.1	6.08	14.8	44.6	−0.232	−17.3
	n	**Sep.1**	9.0	4.6	195.2	4.1	**−3.4**	**10.4**	**28.4**	**−0.185**	**−18.4**	**Sep.1**	9.7	390.6	0.55	6.9	**−3.4**	**10.4**	**28.4**	**−0.185**	**−18.4**
X*LC*	m	Sep.26	6.1	7.2	39.5	5.9	3.6	11.2	9.5	−0.040	−14.6	Sep.6	3.4	306.8	0.25	9.3	9.1	11.0	34.4	−0.181	−15.8
	n	**Sep.1**	5.0	13.9	43.8	9.2	**−3.4**	**10.4**	**28.4**	**−0.185**	**−18.4**	**Sep.1**	9.0	373.3	0.86	10.2	**−3.4**	**10.4**	**28.4**	**−0.185**	**−18.4**
XSK	m	Sep.21	6.3	19.5	44.3	2.2	2.1	14.0	41.9	−0.069	−22.7	Sep.2	0.2	408.7	0.60	10.3	10.3	10.8	34.3	−0.120	−19.7
	n	**Sep.1**	1.2	19.8	27.4	4.0	**−3.4**	**10.4**	**28.4**	**−0.185**	**−18.4**	**Sep.1**	20.7	385.4	0.91	10.3	**−3.4**	**10.4**	**28.4**	**−0.185**	**−18.4**
ELC	m	Mar.3	1.1	7.4	295.2	1.0	−2.3	7.0	40.6	−0.002	−35.8	Oct.19	13.0	267.3	0.29	5.9	5.8	9.5	64.4	−0.208	−5.1
	n	**Sep.1**	6.1	11.2	23.1	0.7	**−3.4**	**10.4**	**28.4**	**−0.185**	**−18.4**	**Sep.1**	32.6	260.7	0.96	9.1	**−3.4**	**10.4**	**28.4**	**−0.185**	**−18.4**
ESB	m	Sep.17	0.9	13.0	18.9	−1.7	−2.9	14.1	20.9	−0.229	−20.5	Oct.20	7.5	560.4	0.16	1.9	1.8	8.5	56.4	−0.250	−4.4
	n	**Sep.1**	2.1	11.1	49.0	1.1	**−3.4**	**10.4**	**28.4**	**−0.185**	**−18.4**	**Sep.1**	49.3	274.8	0.99	−3.1	**−3.4**	**10.4**	**28.4**	**−0.185**	**−18.4**
HAC	m	Oct.16	9.1	11.4	57.0	3.0	−1.6	5.5	52.0	−0.005	−52.7	Sep.5	4.0	358.6	0.45	4.9	4.4	5.4	33.9	−0.158	−14.7
	n	**Sep.1**	10.1	10.4	15.3	4.7	**−3.4**	**10.4**	**28.4**	**−0.185**	**−18.4**	**Sep.1**	30.3	339.4	0.90	8.8	**−3.4**	**10.4**	**28.4**	**−0.185**	**−18.4**
HED	m	Mar.23	7.7	8.3	44.2	2.1	2.0	10.2	59.9	−0.285	−9.0	Oct.7	13.1	236.7	0.08	5.6	0.2	5.8	30.6	−0.795	−7.0
	n	**Sep.1**	10.1	10.1	15.6	4.4	**−3.4**	**10.4**	**28.4**	**−0.185**	**−18.4**	**Sep.1**	20.0	370.1	0.99	9.5	**−3.4**	**10.4**	**28.4**	**−0.185**	**−18.4**

m: all parameters estimated; n: section parameters estimated; *T_low_*: the lowest temperature of the rate of chilling; CU: chilling unit; FU: forcing unit;*T_high_*: the highest temperature of the rate of chilling; *va*, *vb* and *vc*: constants; *T_b_*: base temperature; C*: critical value of state of chilling for the transition from rest to quiescence; F^*^: temperature sum critical threshold; *T_o_*: optimal temperature of the rate of chilling; *K_m_*: minimum potential of unchilled buds to respond to forcing temperature. PSM=*Salix matsudana*; PUP=*Ulmus pumila*; PAV=*Armeniaca vulgaris*; PPS=*Populus simonii*; PSO=*Syringa oblate*; PPNK=*Pinus koraiensis*; PLD=*Larix dahurica*; PPCK=*Picea koraiensis*; XLC=*Leymus chinensis* in Xilinhot; XSK=*Stipa krylovii*; ELC=*Leymus chinensis* in Ewenki; ESB=*Stipa baicalensis*; HAC=*Agropyron cristatum*; HED=*Elymus dahuricus*. The fixed parameters from Chuine et al. (1998) are in bold.

**Table 5:** Root mean square error (*RMSE*), coefficient of determination (*R^2^*) and *F*-test (*F*) for the model fitting to the data.

Species		*RMSE*					*R^2^*					*F*				
		SW	TP	SM	PM	AM	SW	TP	SM	PM	AM	SW	TP	SM	PM	AM
PSM	m	3.33	3.29	1.12	6.13	3.16	0.13	0.13	0.89***	0.01	0.20	1.8	1.79	97.1***	0.12	3.0
	n	2.76	2.76	1.04	0.41	2.50	0.28	0.28	0.90***	0.98***	0.40	4.3	4.3	85.8***	581***	6.6*
PUP	m	3.28	3.28	2.36	2.78	2.81	0.50*	0.50*	0.71***	0.59**	0.63***	10.0*	10.0*	24.5**	14.4**	17.0**
	n	3.19	3.00	1.91	1.35	2.68	0.46	0.54*	0.83***	0.92***	0.64	7.7*	10.4*	43.4***	99.9***	16**
PAV	m	2.20	2.20	1.41	4.40	3.09	0.80***	0.80***	0.94***	0.37	0.82***	40.0***	40.0**	156***	5.9*	45.6**
	n	2.20	2.20	1.38	1.71	2.02	0.80***	0.80***	0.93***	0.91***	0.87***	36.1***	36.1***	122***	87.1***	60***
PPS	m	2.42	2.26	1.67	3.12	2.32	0.62***	0.62**	0.81***	0.36	0.65***	19.6**	19.6**	51.2***	6.8*	22.3**
	n	2.32	2.11	1.24	1.30	2.20	0.62***	0.68***	0.89***	0.88***	0.68***	17.6**	23.4**	91.4***	83.4***	23.2**
PSO	m	1.69	1.89	2.40	6.04	3.11	0.79***	0.76**	0.61**	0.00	0.44*	33.9**	28.5**	14.1**	0.0	7.1*
	n	1.66	1.66	0.39	0.73	2.99	0.79***	0.79***	0.99***	0.97***	0.40	41.3***	40.2***	1066***	333***	7.5*
PPNK	m	3.92	3.92	9.17	2.77	5.85	0.02	0.02	0.07	0.01	0.08	0.10	0.1	0.4	0.05	0.4
	n	2.71	2.97	6.73	0.41	4.62	0.00	0.00	0.10	0.99***	0.04	0.0	0.1	0.5	296***	0.2
PLD	m	2.74	2.45	2.65	5.17	1.68	0.48	0.58*	0.52	0.11	0.77***	4.6	6.9*	5.4	0.6	16.7**
	n	1.15	1.15	0.82	0.91	1.41	0.90***	0.90***	0.96***	0.95***	0.88***	37.4**	37.4**	92.8**	70.6**	30.1**
PPCK	m	1.53	1.78	0.71	0.58	0.82	0.82***	0.75***	0.98***	0.97***	0.95***	22.8**	15.0**	245***	161***	95**
	n	1.29	1.22	0.41	0.41	0.82	0.90***	0.92***	0.99***	0.99**	0.98***	35.5***	44.7***	336***	336***	236***
XLC	m	8.71	3.95	6.03	19.88	7.44	0.01	0.76***	0.73***	0.12	0.14	0.1	28.5**	24.3**	1.2	1.5
	n	8.23	3.58	5.66	5.32	7.28	0.26	0.87**	0.63*	0.57*	0.20	2.8	54.5***	13.8*	10.4*	2.0
XSK	m	5.07	3.96	6.11	25.29	6.02	0.24	0.46*	0.13	0.15	0.29	2.2	6.0*	1.1	1.2	2.9
	n	4.94	3.86	5.04	4.73	6.01	0.57*	0.80***	0.52*	0.66*	0.20	10.8*	32.6***	8.5*	15.4**	2.0
ELC	m	1.90	2.11	2.12	11.47	5.33	0.72***	0.67***	0.91***	0.18	0.04	23.1**	18.3**	91.1***	2.0	0.4
	n	1.60	1.41	1.00	1.05	3.23	0.79***	0.87***	0.93***	0.91***	0.36	26.1**	45.0***	97.8***	69.9***	3.9
ESB	m	3.20	3.14	1.58	12.59	3.32	0.42	0.76***	0.85***	0.37	0.35	5.1	22.2**	39.7**	4.1	3.8
	n	3.04	3.04	0.82	0.75	2.40	0.34	0.34	0.95***	0.96***	0.69**	3.8	3.6	125***	162***	15.3**
HAC	m	6.51	4.91	4.70	11.84	5.88	0.32	0.76***	0.75***	0.18	0.46	3.3	22.2**	21**	1.5	6.0*
	n	4.50	4.06	3.93	3.00	4.29	0.73***	0.80***	0.80***	0.87***	0.74***	18.9**	28.0**	24.8**	40.8***	17.0**
HED	m	6.65	5.94	7.20	11.98	7.67	0.16	0.69***	0.24	0.04	0.06	2.1	24.5**	3.5	0.5	0.7
	n	5.07	4.64	3.48	6.37	6.70	0.34	0.69***	0.80***	0.37	0.16	5.8*	24.7***	39.1***	2.4	1.8

SW: Spring warming model; TP: Temperature-precipitation based leafing model; AM: Alternating model; SM: Sequential model; PM: Parallel model. m: parameterized starting date for SW, TP and AM, all parameters estimated for SM and PM; n: fixed starting date for SW, TP and AM, section parameters estimated for SM and PM. PSM=*Salix matsudana*; PUP=*Ulmus pumila*; PAV=*Armeniaca vulgaris*; PPS=*Populus simonii*; PSO=*Syringa oblate*; PPNK=*Pinus koraiensis*; PLD=*Larix dahurica*; PPCK=*Picea koraiensis*; XLC=*Leymus chinensis* in Xilinhot; XSK=*Stipa krylovii*; ELC=*Leymus chinensis* in Ewenki; ESB=*Stipa baicalensis*; HAC=*Agropyron cristatum*; HED=*Elymus dahuricus*.

* : *P*<0.05;

** : *P*<0.01;

*** : *P*<0.001.

## Results

### Model fitting

The model comparison would be evaluated by *RMSE*, coefficients of determination (*R* ^2^) and sum of residual squares (e.g., *F*-test). The average *RMSE* for all models fitting ranged from 2.00 to 4.18. The average *RMSE*s of PM with all parameters estimated (PMm) was the smallest, and the average *RMSE*s of SM with all parameters estimated (SMm) was close behind (Tables 3, 4, 5). However, the result of models fitting did not represent an accurate model test. The model validation would be assessed using independent data.

The average *RMSE*s of temperature - precipitation based leafing model (TP) with fixed and parameterized starting date were 3.22 and 2.69 days, respectively. *k_1_* (average=0.05) of TP was smaller than *k_2_* (average=0.45) (Table 3). The hydrological conditions for plant leafing varied with available water and climate in different regions. Thus, model coefficients comprehensively reflected the hydrological requirements for plant leafing. The mean hydrological requirement for woody plants was more than herbs in Northeast China.

### Model validation

**Figure 2:**
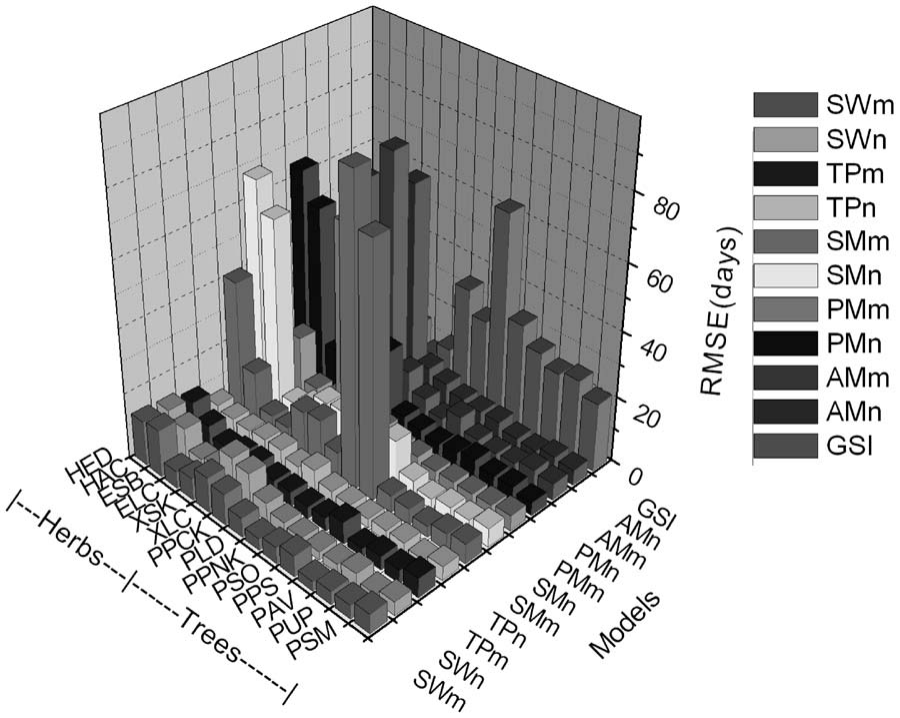
The root mean square error (*RMSE*) of different phenology models for plant leafing using independent data. Tree species include PSM=*Salix matsudana*; PUP=*Ulmus pumila*; PAV=*Armeniaca vulgaris*; PPS=*Populus simonii*; PSO=*Syringa oblate*; PPNK=*Pinus koraiensis*; PLD=*Larix dahurica*; and PPCK=*Picea koraiensis*. Herbs include XLC=*Leymus chinensis* in Xilinhot; XSK=*Stipa krylovii*; HED=*Elymus dahuricus*; HAC=*Agropyron cristatum*; ELC=*Leymus chinensis* in Ewenki; and ESB=*Stipa baicalensis*. SWn, TPn, and AMn: Spring warming model (SW), Temperature-precipitation based leafing model (TP), and Alternating model (AM) with fixed starting date. SWm, TPm, and AMm: SW, TP and AM with parameterized starting date. SMn and PMn: Sequential model (SM) and Parallel model (PM) with section parameters estimated. SMm and PMm: SM and PM with all parameters estimated. GSI: Growth season index.

**Figure 3:**
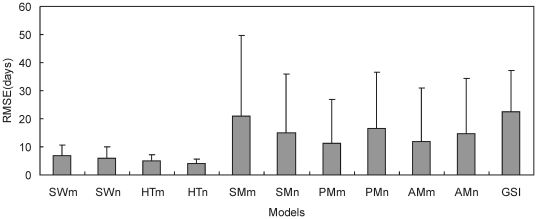
Average root mean square error (*RMSE*) of different phenology models for plant leafing using independent data. SWn, TPn, and AMn: Spring warming model (SW), Temperature-precipitation based leafing model (TP), and Alternating model (AM) with fixed starting date. SWm, TPm, and AMm: SW, TP and AM with parameterized starting date. SMn and PMn: Sequential model (SM) and Parallel model (PM) with section parameters estimated. SMm and PMm: SM and PM with all parameters estimated. GSI: Growth season index.

We validated these models using plant leafing data of even-numbered years in Northeast China. TP with fixed starting date (TPn) had the smallest average *RMSE* (4.21 days). The *RMSEs* of TPn for all plant species ranged from 2.22 to 6.44 days. The average *RMSE* of TP with parameterized starting date (TPm) was 5.12 days. The average *RMSE* of SW with fixed starting date (SWn) was smaller than SW with parameterized starting date (SWm) (i.e., 6.09 days <6.95 days). The average *RMSEs* of the other models were more than 10 days (Fig. 2, Fig. 3).

For tree leafing, *R* ^2^ was greater than 0.60 for 75% of the estimates by SWn and TPn, but less than 50% for the others. Both SWn and TPn had *RMSE* less than five days in 87.5% of the estimates, while they met this criterion less than 75% of the time (Table 6). Based on *R* ^2^ and *RMSE*, SWn and TPn were the best models for simulating tree leafing in Northeast China.

**Table 6:** Coefficient of determination (*R* ^2^) of model verification using independent data for the plant leafing in Northeast China.

Species		SW	TP	SM	PM	AM	GSI
PSM	m	0.53**	0.56**	0.69***	0.76***	0.58**	0.47*
	n	0.67***	0.66***	0.72***	0.72***	0.67***	
PUP	m	0.58**	0.66***	0.31	0.30	0.23	0.59**
	n	0.82***	0.80***	0.28	0.26	0.28	
PAV	m	0.65***	0.64***	0.73***	0.69***	0.69***	0.61**
	n	0.64***	0.65***	0.66***	0.57**	0.77***	
PPS	m	0.74***	0.80***	0.52**	0.56**	0.57**	0.46*
	n	0.74***	0.72***	0.79***	0.24	0.53	
PSO	m	0.23	0.22	0.37	0.76***	0.64***	0.79***
	n	0.84***	0.82***	0.46*	0.58**	0.45*	
PPNK	m	0.05	0.13	0.01	0.31	0.20	0.72**
	n	0.45	0.42	0.00	0.01	0.35	
PLD	m	0.92***	0.92***	0.00	0.60*	0.70**	0.94***
	n	0.96***	0.97***	0.96***	0.58*	0.48	
PPCK	m	0.14	0.48	0.14	0.21	0.22	0.24
	n	0.34	0.24	0.33	0.29	0.15	
XLC	m	0.04	0.80***	0.06	0.17	0.11	0.00
	n	0.02	0.82***	0.00	0.37	0.10	
XSK	m	0.11	0.68**	0.03	0.08	0.00	0.00
	n	0.33	0.69**	0.06	0.45*	0.21	
ELC	m	0.09	0.58*	0.62**	0.52*	0.32	0.06
	n	0.02	0.40*	0.23	0.17	0.02	
ESB	m	0.22	0.50*	0.47*	0.37	0.46*	0.18
	n	0.51*	0.53*	0.54*	0.09	0.51*	
HAC	m	0.02	0.69**	0.01	0.18	0.00	0.48*
	n	0.03	0.80***	0.01	0.13	0.00	
HED	m	0.01	0.18	0.08	0.01	0.07	0.04
	n	0.00	0.81***	0.01	0.09	0.05	

SW: Spring warming model; TP: Temperature-precipitation based leafing model; SM: Sequential model; PM: Parallel model; AM: Alternating model; GSI: Growth season index. m: parameterized starting date for SW, TP and AM, all parameters estimated for SM and PM; n: fixed starting date for SW, TP and AM, section parameters estimated for SM and PM. PSM=*Salix matsudana*; PUP=*Ulmus pumila*; PAV=*Armeniaca vulgaris*; PPS=*Populus simonii*; PSO=*Syringa oblate*; PPNK=*Pinus koraiensis*; PLD=*Larix dahurica*; PPCK=*Picea koraiensis*; XLC=*Leymus chinensis* in Xilinhot; XSK=*Stipa krylovii*; ELC=*Leymus chinensis* in Ewenki; ESB=*Stipa baicalensis*; HAC=*Agropyron cristatum*; HED=*Elymus dahuricus*.

* : *P*<0.05;

** : *P*<0.01;

*** : *P*<0.001.

When we validated the models using even-year data for herb leafing, TPn model yielded *RMSE* of 4–6 days (average 5.03 days) and *R* ^2^ of 0.4–0.82 (average 0.675); followed by TPm with average *RMSE* and *R* ^2^ of 6.21 days and 0.572, respectively. For the other models, the minimum and average *RMSE* were more than 4.50 and 9.51 days, respectively (Fig. 2), and the maximum and average *R* ^2^ were less than 0.62 and 0.22, respectively (Table 6). Consequently, TPn was the best model for simulating herb leafing in the present study.

## Discussion

**Figure 4:**
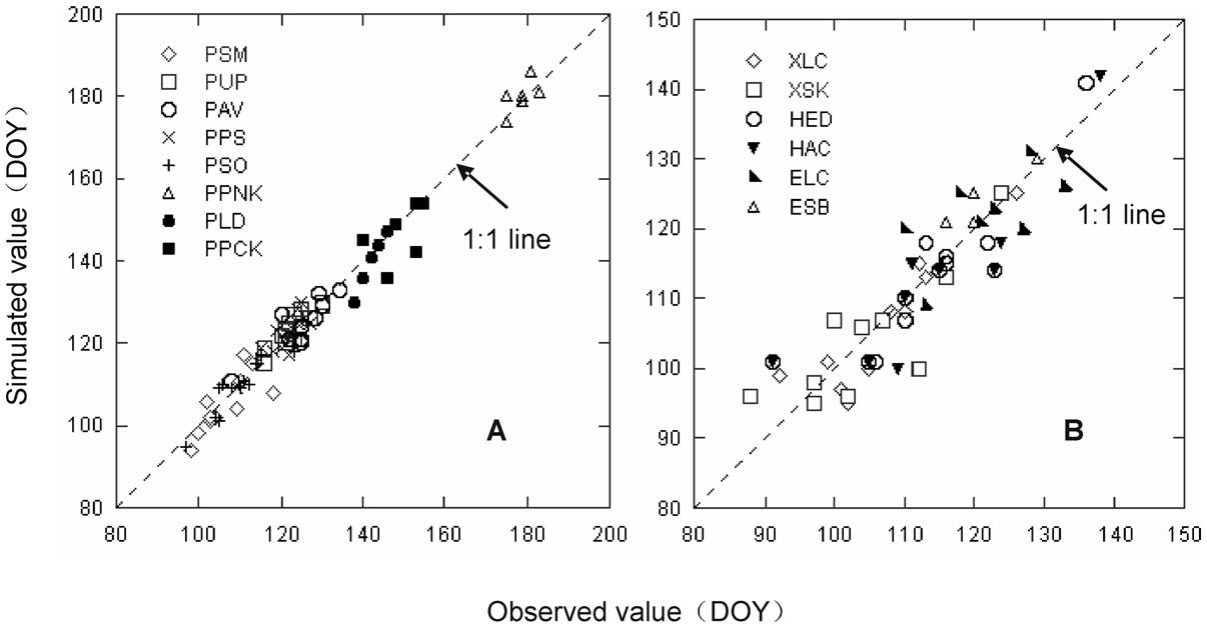
Observed versus simulated values for tree (A) and herb (B) leafing in Northeast China based on Temperature-precipitation based leafing model (TPn). Tree species include PSM=*Salix matsudana*; PUP=*Ulmus pumila*; PAV=*Armeniaca vulgaris*; PPS=*Populus simonii*; PSO=*Syringa oblate*; PPNK=*Pinus koraiensis*; PLD=*Larix dahurica*; and PPCK=*Picea koraiensis*. Herbs include XLC=*Leymus chinensis* in Xilinhot; XSK=*Stipa krylovii*; HED=*Elymus dahuricus*; HAC=*Agropyron cristatum*; ELC=*Leymus chinensis* in Ewenki; and ESB=*Stipa baicalensis*.

Previous works have suggested that *R* ^2^ [28] or *RMSE* [29] are better than other criteria for comparing different nonlinear models. Generally, the smaller the *RMSE* value, the larger the *R* ^2^ value. However, the opposite case (i.e., a smaller *RMSE* was associated with a smaller *R* ^2^) occasionally appeared in the model calibration of SW and TP for leafing of *Salix matsudana* (Table 5). SWn could simulate the leafing of *Salix matsudana* precisely (*R* ^2^=0.67, *P*<0.001) (Table 6). This result supports the opinion that *RMSE* is a more reliable measure of fit than *R* ^2^ for nonlinear regression [29]. The opposite case may be due to the noise of parameterized data for *S. matsudana*. Furthermore, the *RMSE* of plant leafing in Northeast China given by TPn ranged from 2.22 to 6.44 days, and the predicted DOY values for leafing were close to the observed values (Fig. 4). It indicated that TP can precisely simulate the leafing stages of both woody plants and herbs in Northeast China.

The model accuracy for tree leafing at high latitudes could not necessarily be improved with more complex models, consistent with the result of Hannerz [30]. In the present study, the most complicated model, PM, could give better simulation with the least precise, while a much simpler model, SW, with more accurate. SM, AM, and PM consider all the factors in the chilling process, whereas SW and TP do not, i.e., the former models include more information on temperature change through time during parameterization process. However, these three models (SM, AM, and PM) performed poorly when validated using actual observed data at different times, as seen with *Ulmus pumila* (Table 6).

SW and TP without chilling requirement considering the starting date can reflect the effect of temperature beyond base temperature at different periods on plant leafing. Models with both fixed starting date (SWn and TPn) on 1 January and parameterized starting day (SWm and TPm) were analyzed in the present study. We found that after the validation by independent data, SWn (average *RMSE* of 6.05 days) could give better leafing simulation than SWm (average *RMSE* of 6.95 days), and TPn (average *RMSE* of 3.59 days) was better than TPm (average *RMSE* of 4.31 days). This result indicated that the temperature beyond base temperature at most periods could affect plant leafing. Generally, the models with parameterized starting date can consider only the temperature beyond base temperature after starting date, but the temperature before starting date might have important effect on plant leafing. In Northeast China, the coldest month is January with mean air temperature of −18.2°C. Compared with SWm and TPm, SWn and TPn can give better simulation in Northeast China because the effective temperature during longer period can be considered. According to the observation data, tree leafing tended to advance with the extent of 0.23 days yr^−1^ during 1980–2005, and was significantly negatively correlated with temperature in February, March and April. Furthermore, the effect of average temperature in April and February on plant leafing was the largest (2.35 days °C^−1^) and the smallest (1.18 days °C^−1^), respectively [31]. Therefore, regarding to climate warming, the main driving factor of plant leafing should be temperature instead of light [32], and SWn and TPn could give better simulation of plant leafing.

The simulation precision of phenology models with the consideration of chilling is critically influenced by the starting date. In Europe, phenology models are set to start on 1 September [23] or 1 November [7], [10]. In this study, SMn, PMn and AMn were set to start on 1 September and the starting dates of SMm, PMm and AMm were parameterized using odd-year data. There are larger *RMSEs* for those models from independent data, and the reasons are attributed to the starting date: (1) the starting date is set early, resulting in untimely meeting the chilling and forcing thermal requirements. For example, the *RMSE* of SM with starting date on 1 September and 16 October for *Agropyron cristatum* were 58.9 and 11.57 days, respectively (Table 4, Fig. 3); and (2) the starting date is set late, leading that chilling thermal requirement can not meet. e.g., the *RMSE* of SM with starting date on 1 September was smaller (3.87 days) than 13 November (92.33 days) for *Larix dahurica* (Table 3, Fig. 3).

The fixed parameters (*t_0_*, *T_low_*, *T_high_*, *va*, *vb* and *vc*) in SM and PM, from the result of Europe [9], [11], [12], [16], were widely used in plant phenology models [10], [21], [23]. Furthermore, these parameters were estimated using local observing data [31]. In the model fitting process of the present study, all thresholds and parameters of SM and PM were estimated. We found that the models with many parameters could be fitted well (Tables 3, 4, 5), but could not give accurate simulation when tested with the independent data (Fig. 2). This finding was consistent with the result from Linkosalo et al. [33], and it might be because the models were over-parameterized and able to adapt to noise in addition to the phenological phenomenon itself [33]. The parameter estimate lies on the parameter space boundary [34].

Variation in the base temperature has no significant effect on the precision of spring phenology models [14], [30], [35]. Generally, the heat unit total depends on the threshold used [35], as with *Leymus chinensis* in this study (Tables 3, 4, 5). Therefore, changes in the base temperature induce different thresholds for the accumulated temperature, resulting in no significant variation in model accuracy. The threshold measure is a mathematical construct which may or may not be related to the physiological threshold [34]. Physiological parameters can be estimated from simulation experiments, but can not be obtained from the process of parameter optimization. This is because the optimization process is mostly dependent on the precision of observed field data, sample number, and local climate conditions. The biological interpretation of model parameters should not be considered as absolute [34], [36]. The base leafing temperature of the same plant can be different simply due to different models, as seen with all plant species in this study (Tables 3, 4, 5). The base temperature in the growth season index (GSI) is fixed, and the minimum temperature is derived from experimental data [36], [37]. In the present study, the leafing dates of woody plants actually changed can be, to a certain extent, explained by GSI using the fixed parameter. However, considering the threshold of 0.5 from the original model [25], the predicted dates for plant leafing in Northeast China was earlier than the observed values. Thus, the original parameter threshold for GSI was too small for the present study, and the optimal threshold varied with different species.

Previous studies indicated that the phenology of woody plants in temperate regions can be accurately predicted by a temperature-based model (e.g., SW). For example, SW is considered to be the best model to accurately simulate the bud development of *Picea abies* [30] and has been validated [23]. Chilling has been introduced into some temperature-based models (e.g., AM) to improve accuracy. For example, AM is much more suitable for simulating budburst of *Picea abies* [7]. Furthermore, there is a correlation between chilling and forcing, i.e., forcing takes the effect after the chilling has finished [38].

In this study, plant leafing in Northeast China was simulated using four temperature-based and two temperature-precipitation based phenology models. When validated with independent data, SW and TP could give best simulation of the woody plant leafing. The effect of temperature in TP was the same with SW, and its accuracy was consistent with SW in moist conditions. The effect of precipitation in TP does not change the model manner of temperature, therefore (1) TP with fixed starting date (TPn) could be used to simulate the leafing affected by hydrological factors. For example, leafing of *Leymus chinensis* and *Stipa krylovii* in Xilinhot, Inner Mongolia was delayed 27 and 22 days due to water stress in some years; in other locations, the average delay time for *Leymus chinensis*, *Stipa baicalensis*, *Elymus dahuricus* and *Agropyron cristatum* reached 7, 5, 14 and 16 days, respectively; and (2) the leafing of woody plants in Northeast China was mainly driven by thermal conditions, and hydrological conditions were not limiting factors. For example, the average *RMSE* of SWn was much close to TPn for tree leafing (Fig. 2). This finding was consistent with other studies in which the precision simulating the leafing of broad-leaved deciduous plants could not be substantially improved by adding precipitation into the model [26]. Kramer et al. [39] also believed that, in temperate zone, water availability mainly affects leaf area index and has little effect on leaf phenology. However, different species have different sensitivities to water conditions, and the leafing of some trees may be affected by hydrological conditions. Thus, TP is suitable for a wider range of plants because it considers the effects of both temperature and precipitation on plant leafing.

**Figure 5:**
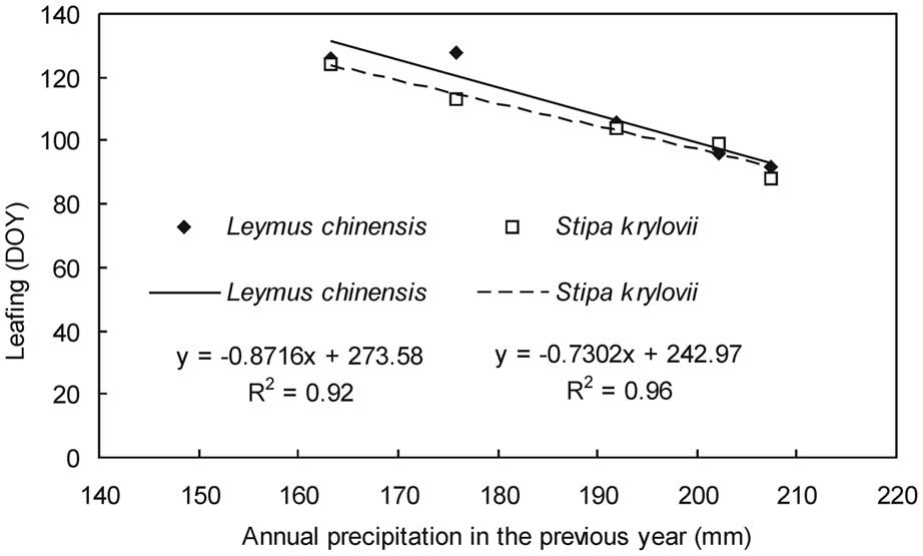
Relationship between annual precipitation in previous year and leafing of two herbs (i.e., *Leymus chinensis* and *Stipa krylovii*) in Xilinhot, Inner Mongolia.

TP was far superior to other five models in estimating herb leafing in Northeast China, and all simulated dates were very close to the observed dates (Fig. 4B). Other five models can not accurately simulate herb leafing. In addition to temperature, water availability was shown to be an important controlling factor for the phenology of herbs [18]. For example, the annual precipitation of 5 years (1984, 1989, 2000, 2001, and 2002) was less than 210 mm in Xilinhot, and was negatively correlated with herb leafing in next year (*R* ^2^>0.9; Fig. 5). Therefore, the reduction of previous annual precipitation leads to the delay of herb leafing. It is reasonable to consider the effect of previous annual precipitation in TP. Because herbs generally have shallow roots, they tend to be strongly affected by hydrological conditions. Herb leafing in response to environmental conditions can be estimated using hydrological factors incorporated into the TP model. Although GSI can model the effects of hydrological factors with vapor pressure deficit (VPD), the validation of the model was rather poor in the present study, due to the lack of available VPD value or the lower sensitivity of VPD to hydrological conditions (Fig. 4). In addition, two instances can indicate that TP is superior over SW in more arid areas: (1) the *RMSE* of TPn was smaller than that of SWn for *Leymus chinensis* and *Stipa krylovii* in arid Xilinhot, and (2) the predicted leafings from SWn and TPn with the same *T_b_* and F* were very close in moist years, and much closer to the observed value from TPn in drought years than SWn (data not shown).

Phenology model parameters can be obtained experimentally, but most phenology models use parameters optimized based on long-term observed data, e.g., the four temperature-based models (SW, SM, PM, and AM) and the temperature-precipitation based leafing model (TP). Different parameters are selected for different plant species in various regions. Therefore, sufficient data should be used to ensure the effectiveness and reliability of the model parameters. In this study, leafing simulations of *Pinus koraiensis* and *Picea koraiensis* were poor because the parameters were optimized based on only six years' observed data (Table 6, Fig. 2). There is a strong possibility that more errors occur when the model is based on less data. However, the parameters of leafing phenology models for the other six woody plants were optimized using 12 years' observed data, and all models accurately simulated plant leafing. Overall, TP will be more suitable and reliable for modeling both woody and herbaceous plant leafing given climate changes (especially variation in hydrological conditions), while other leafing models that do not consider water will be less applicable in semi-arid and arid areas.
